# Eicosapentaenoic acid inhibits endothelial cell migration *in vitro*

**DOI:** 10.1186/2040-2384-2-12

**Published:** 2010-07-09

**Authors:** Laura Tonutti, Luca Manzi, Maria T Tacconi, Gianfranco Bazzoni

**Affiliations:** 1 Mario Negri Institute of Pharmacological Research, Via La Masa 19, Milano, Italy

## Abstract

**Background:**

As *n*-3 Polyunsaturated Fatty Acids exert a beneficial action on the cardiovascular system, it is important to investigate their effects on endothelial cell responses that (like migration) contribute to repairing vascular lesions.

**Methods:**

To this purpose, using functional and morphological *in vitro *assays, we have examined the effect of *n*-3 Polyunsaturated Fatty Acids on the migration of endothelial cells.

**Results:**

We report here that incubation of endothelial cells with *n*-3 Polyunsaturated Fatty Acids impaired cell migration into a wound, triggered peripheral distribution of focal adhesions and caused partial disassembly of actin filaments. We also found that eicosapentaenoic acid and docosahexaenoic acid exerted similar effects on the focal adhesions, but that eicosapentaenoic acid was sufficient for inhibiting cell migration.

**Conclusions:**

Given the importance of endothelial cell migration in the repair of vascular injuries, these *in vitro *findings call for *in vivo *evaluation of vascular repair in response to different dietary ratios of eicosapentaenoic to docosahexaenoic acid.

## Background

Dietary intake of *n*-3 Polyunsaturated Fatty Acids (PUFA) is perhaps the most favourable lipid-lowering intervention that is associated with reduced risks of mortality [1]. In particular, a randomized clinical trial supports their use after myocardial infarction [2]. In addition, *n*-3 PUFA exert a protective action against general diseases that affect the cardiovascular system, such as obesity and diabetes [1]. Therefore, detailed analysis of the effects of *n*-3 PUFA on vascular cells is expected to provide a more rational basis for their clinical use. However, our understanding of the effects of *n*-3 PUFA at the cellular level is far from being complete.

In this study, we focused on the effect of *n*-3 PUFA on endothelial cell (EC) migration, because migration is essential for vessel formation (*e.g*. collateral growth in ischemia) and repair (*e.g*. endothelial healing upon angioplasty). In this respect, the *n*-3 PUFA eicosapentaenoic acid (EPA) and docosahexaenoic acid (DHA) are of particular interest, not only because they are widely used in cardiovascular medicine [1], but also because they change the fatty acid composition of the membranes [3] and affect different types of membrane proteins. In particular, by modulating the relative phospholipid composition of the lipid rafts [4,5], *n*-3 PUFA affect the association of some signaling proteins with the lipid rafts [6,7].

Furthermore, *n*-3 PUFA regulate gene expression [8], modulate eicosanoid activity [3] and exert variable effects on other signaling pathways, including Akt and Erk-1/2 phosphorylation [9]. Concerning endothelial cells, studies aimed at explaining the protective action of *n*-3 PUFA in atherosclerosis and thrombosis have reported on the ability of DHA to inhibit endothelial responses to inflammatory cytokines, including the expression of adhesion molecules involved in leukocyte migration [10] and the induction of cycloxigenase-2, which ultimately may trigger NFκB activation [11].

Here, we surmised that, in addition to these responses, PUFA might interfere with other biological processes that involve membrane proteins, such as cell migration.

## Results

### Addition of *n*-3 PUFA increases the membrane content of EPA and DHA

**Figure 1:**
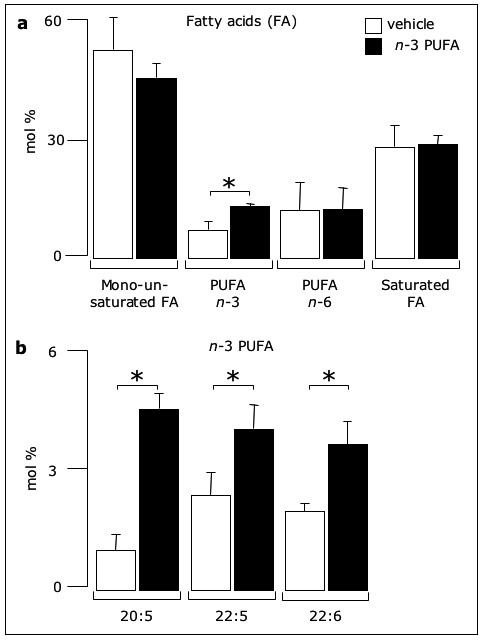
***n*-3 PUFA change the relative fatty acid composition of endothelial membranes**. Confluent EC were incubated for 24 hours with either ethanol (*vehicle*) or 50 μM *n*-3 PUFA EE. Then, membranes were analyzed by chromatography for the composition of (a) total *n*-3 PUFA, *n*-6 PUFA and saturated fatty acids, as well as (b) the *n*-3 PUFA 20:5, 22:5 and 22:6. Results are mean ± SD from three experiments (* p < 0.05).

Incubation of confluent EC (for 24 hours at 37°C) with a mixture of *n*-3 PUFA ethyl esters (EE) caused a significant increase in the membrane content of total *n*-3 PUFA (*i.e*. the sum of 20:5, 22:5 and 22:6 *n*-3) and a slight decrease in monounsaturated fatty acids, while no changes in *n*-6 PUFA and saturated fatty acids were detectable (Fig. 1a). More specifically, the content of EPA (20:5), its elongation product (22:5) and DHA (22:6) were all significantly increased compared to vehicle (ethanol)-incubated cells (Fig. 1b). Other *n*-3 PUFA (*e.g*. 18:3 and 20:3) were undetectable in both vehicle-and *n*-3 PUFA-incubated cells.

### *n*-3 PUFA inhibit endothelial cell migration

**Figure 2:**
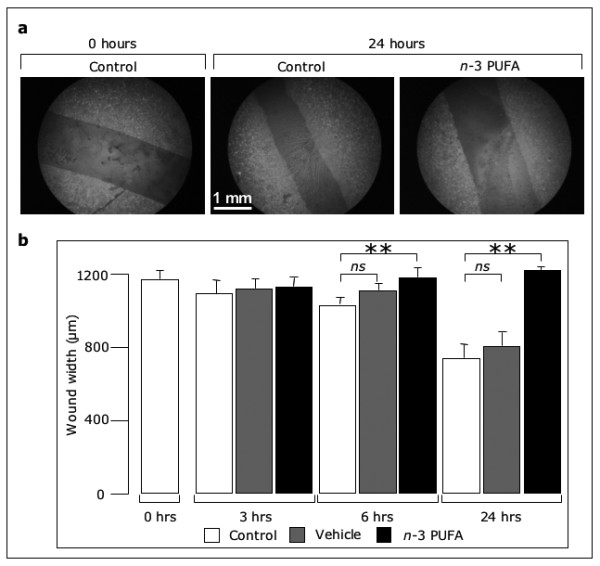
***n*-3 PUFA inhibit migration into a wound**. Confluent EC were wounded and incubated with medium, vehicle or *n*-3 PUFA EE. Then, cells were fixed, stained with FITC-phalloidin and examined by fluorescence microscopy. Representative samples are shown in (a), while the wound widths are reported in (b) as mean ± SD from three experiments performed in duplicate (** p < 0.01).

We next examined the effect of *n*-3 PUFA on endothelial cell migration. To this purpose, confluent EC monolayers were scratch wounded and then incubated with medium (control), ethanol (vehicle) or 100 μM *n*-3 PUFA EE for different time points (0, 3, 6 and 24 hours). Finally, the width of the wound was measured to quantify migration (Fig. 2a). In control-and vehicle-treated cells, the wound width was reduced at 6 and 24 hours, compared with the initial width (at time 0). In contrast, in *n*-3 PUFA-treated cells, the width was not reduced, thus indicating that *n*-3 PUFA impaired migration into the wound (Fig. 2b). Cell viability, as evaluated by the trypan blue exclusion test, was not significantly different between cells that had been treated (for 24 hours) with either vehicle or 100 μM PUFA EE (79 ± 8% and 76 ± 4% of viable cells, respectively).

### EPA is responsible for the *n*-3 PUFA effect on cell migration

The mixture of *n*-3 PUFA EE, which was used for the above experiments, contains EPA EE (46.2%), DHA EE (40.8%) and other fatty acid EE (13.0%). To define which of the major *n*-3 PUFA was responsible for the inhibition of migration, we used purified EPA EE and DHA EE, either alone or in combination, and then measured the wound width (Table 1).

**Table 1:** Specificity of the *n*-3 PUFA effect on cell migration

***Treatment***	***Wound width (μm)***
Medium	852 ± 15

Vehicle (ethanol)	905 ± 118 *ns*

PUFA EE	1166 ± 94 **

DHA EE	896 ± 51 *ns*

EPA EE	1189 ± 60 **

DHA EE + EPA EE	1156 ± 9 **

Confluent EC monolayers were wounded and treated with the indicated compound (each at a concentration of 100 μM) for 24 hours before being fixed and inspected by fluorescence microscopy to quantitate the wound width. The initial width (at time 0) measured 1194 ± 7 μm. Data are means ± SD from three different experiments (each performed in duplicate). **p < 0.01; *ns *not significant (compared with medium).

As above, at 24 hours, the wound width was reduced in control- and vehicle (ethanol)-treated cells, but not in cells treated with the mixture of *n*-3 PUFA EE. In addition, we found that the wound width was reduced in cells treated with DHA EE alone, but not with EPA EE (either alone or in combination with DHA EE), thus suggesting that EPA was responsible for the anti-migratory action.

### *n*-3 PUFA-dependent inhibition of migration is MAPK-independent

**Figure 3:**
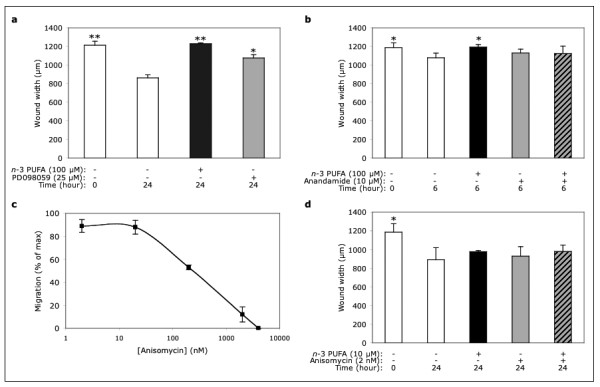
**Role of MAPK in the *n*-3 PUFA-dependent inhibition of migration**. At 24 hours after plating, EC monolayers were switched to low-serum (1%) medium and pre-treated with the indicated MAPK regulators for additional 24 hours. Finally, monolayers were wounded and incubated (in low-serum medium) in either the absence or presence of the indicated compounds for either 6 (b) or 24 (a, c and d) hours. Data are mean ± SD from three experiments, each performed in duplicate (* p < 0.05; ** p < 0.01, compared with the control treatment at the same time point, *second column bar*).

Mitogen-Activated Protein Kinases (MAPK) are key regulators of cell migration [12]. In particular, EC migration into a wound requires ERK-1/2 activation [13]. In agreement with these findings, we observed that migration was reduced in cells treated with the ERK-1/2 inhibitor PD098059, compared with control-treated cells (Fig. 3a). However, when analyzing cells that had been treated in either the presence or absence of the *n*-3 PUFA EE mixture, EPA EE and DHA EE (either alone or in combination, each at 100 μM), we found that none of the treatments changed the levels of total and active ERK-1/2, as assessed by Western blotting (not shown). In addition, the ERK-1/2 activator anandamide neither increased migration *per se *nor prevented the *n*-3 PUFA-dependent inhibition of migration (Fig. 3b). Anandamide effect was evaluated at 6 hours, as it was toxic at longer incubation times.

In addition, we found that the p38/SAPK2 activator anisomycin induced dose-dependent (2-2,000 nM) inhibition of migration, with half-maximal inhibition at about 250 nM (Fig. 3c). However, non-inhibitory concentrations of *n*-3 PUFA (10 μM) and anisomycin (2 nM) did not potentiate each other (Fig. 3d). Thus, although cell migration was sensitive to either ERK-1/2 inhibition or p38/SAPK2 activation, it is conceivable that *n*-3 PUFA did not inhibit migration by regulating the activation state of these two MAPK.

### *n*-3 PUFA-dependent inhibition of migration is associated with cytoskeletal changes

**Figure 4:**
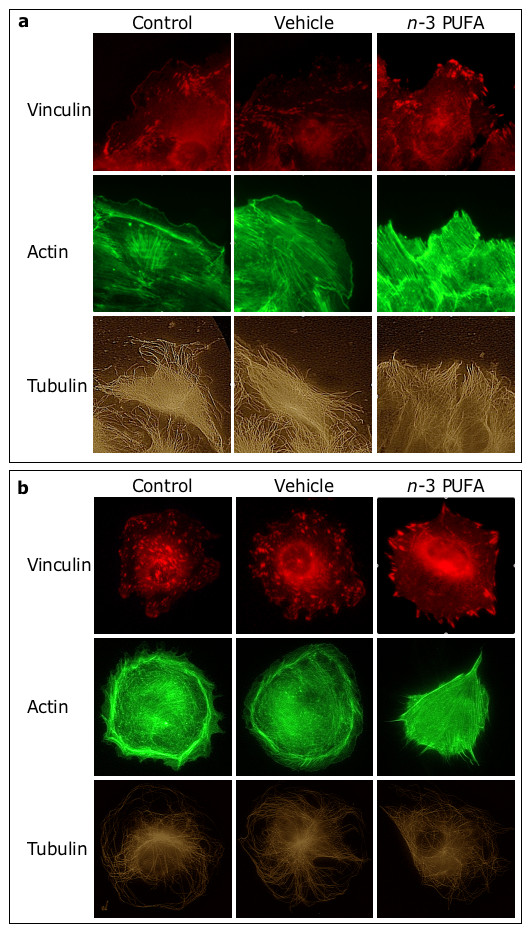
***n*-3 PUFA affect FA and actin filaments**. In (a), EC were grown to confluence and wounded. In (b), EC were allowed to adhere (as non-contacting cells) for 2 hours. Then, cells were incubated for 24 hours (a) or 1 hour (b) with medium (*control*), ethanol (*vehicle*) and 100 μM *n*-3 PUFA EE.

Fluorescence microscopy revealed morphological differences in the migrating cells that were facing the wound (Fig. 4a). Specifically, in control-and vehicle-treated cells, vinculin-containing focal adhesions (FA) distributed evenly at the base of the leading lamella, while actin filaments aligned in parallel to the wound. In contrast, in *n*-3 PUFA-treated cells, FA localized to the free cell edge, actin filaments aligned perpendicular to the wound, and the number of cells that extended lamella-like protrusions was reduced.

Similar cytoskeletal changes were detectable in the confluent cells that were not facing the wound (not shown) and in non-contacting cells (Fig. 4b). Specifically, in non-contacting cells incubated with medium or vehicle, FA evenly distributed throughout the cell, while actin filaments organized in cortical fibers. In contrast, upon treatment with *n*-3 PUFA EE, FA localized almost exclusively to the cell periphery, while cortical actin was replaced by thin protrusions. Notably, *n*-3 PUFA did not affect microtubule morphology in contacting and non-contacting cells alike.

We also determined that the fraction of non-contacting cells with peripheral FA was negligible in control- and vehicle-treated cells (0.0 ± 0.0 and 0.7 ± 1.2%, respectively), but high (70.0 ± 10.6%) in cells treated with the mixture of *n*-3 PUFA EE. A significant fraction was also detected in cells treated with EPA EE (24.0 ± 11.1%), DHA EE (17.7 ± 5.5%) or the combination of both EPA and DHA EE (67.0 ± 15.0%). Thus, FA distribution was attributable to the combined action of EPA and DHA.

## Discussion

The major findings of this study are that *n*-3 PUFA (i) inhibited migration into a wound and (ii) induced rearrangements of migration-related cytoskeletal structures (*i.e*. FA and actin filaments). In addition, (iii) while EPA and DHA exerted similar effects on the FA, EPA was sufficient for inhibiting migration.

The anti-migratory effect of EPA was not expression of a nonspecific anti-motility action. Actually, using the modified Boyden chamber assay, previous studies reported that EPA (but not DHA) increased EC migration [14] and rescued the anti-migratory action of cholesterol [15]. These studies are not at odds with the herein reported inhibitory action of EPA, as the Boyden assay analyzes the individual migration of single cells, while the wound assay evaluates the collective migration of cohesive cell sheets. In addition, our findings mirror the observation that EPA (but not DHA) inhibited the ability of EC to form tube-like structures in collagen gels, which requires coordinated motility and cytoskeletal rearrangements of cohesive EC [16].

Published evidence prompted us to evaluate the possible involvement of the MAPK. In particular, hydrogen peroxide-dependent distribution of the FA to the cell periphery [17] (similar to the one induced by *n*-3 PUFA) required p38/SAPK2 activation and/or ERK-1/2 inhibition [18,19]. Notably, also *n*-3 PUFA were reported to induce and reduce the activation state of p38/SAPK2 [20] and ERK-1/2 [21,22], respectively. In line with these observations, we found that a p38/SAPK2 activator and an ERK-1/2 inhibitor alike impaired EC migration. However, the use of *n*-3 PUFA and MAPK modulators indicated that the herein reported effect of the *n*-3 PUFA on migration was MAPK-independent. Thus, the molecular identity of the mediator(s) of *n*-3 PUFA action deserves further investigation.

Most of our experiments relied on the use of a mixture of *n*-3 PUFA EE, which is also used as dietary supplement for laboratory animals, thus allowing comparative analysis of *in vitro *and *in vivo *data. The herein reported anti-migratory action of *n*-3 PUFA *in vitro *has a potential interest for cardiovascular pharmacology, as it suggests that *n*-3 PUFA might delay wound repair in areas of endothelial damage (*e.g*. atherosclerotic plaques). In addition, the impairment in endothelial repair might explain why dietary supplementation with *n*-3 PUFA does not exert significant prevention of atherosclerotic plaque progression in carotid arteries [23].

However, we also used purified *n*-3 PUFA EE, which provided us with further insights. First, the ability of EPA (20:5), but not DHA (22:6), to inhibit cell migration suggests that the effect is related to the length of the carbon chain. Similarly, in T cells, EPA, but not DHA, inhibited signaling downstream of Src-like tyrosine kinases, which might be due to differences in the ability of the two PUFA to incorporate into membrane microdomains and to displace these signaling molecules [24]. Thus, it is possible that also the EPA-specific inhibition of migration is due to specific interactions with the membrane microdomains. Second, the use of purified *n*-3 PUFA EE has allowed us to determine that both EPA and DHA inhibited FA distribution, but that EPA was sufficient to inhibit migration. Clearly, although FA formation is a key step of migration, the process of migration involves additional responses (*e.g*. lamellipodia extension and actin-myosin contraction) that might be more sensitive to EPA than to DHA.

Other studies have shown differences in the biological effects of EPA and DHA [25]. In addition to differences in metabolic and hemodynamic effects (*e.g*. dyslipidemia improvement and blood pressure reduction), EPA and DHA differ in their ability to reduce inflammatory and thrombotic responses. In particular, emerging evidence indicates that DHA is more efficient than EPA in inhibiting monocyte activation and platelet aggregation. For instance, DHA (but not EPA) decreased cytokine production, the expression of endothelial adhesion molecules and monocyte adhesion to the EC [26]. In addition, DHA was more effective than EPA in reducing platelet aggregation and thromboxane release [27]. Thus, together with these findings, our observations may have practical implications. With all the caveats of transferring experimental data to the clinical practice, we suggest that decreasing the dietary supplementation of EPA might prove beneficial in acute conditions of vascular damage (*e.g*. following angioplasty), not only because DHA is more potent than EPA in inhibiting platelet aggregation and local inflammation, but also because (as shown here) DHA does not interfere with the ability of EC to repair areas of endothelial damage.

Clearly, *in vivo *assays are necessary to validate the *in vitro *effect of *n*-3 PUFA on wound re-endothelialization. However, the available *in vivo *assays, such as arterial injury after balloon angioplasty [28], do not exactly mirror our *in vitro *situation. In particular, the most commonly documented response to angioplasty is hyperplasia of the intima [29] and not endothelialization of the wound. In addition, in the fewer studies that reported endothelial regeneration, the response was more dependent on the recruitment of bone marrow-derived EC progenitors than on the migration of EC from the wound edge [30,31]. Interestingly, our findings are reminiscent of *in vivo *effects of *n*-3 PUFA in the skin excision assay [32], even though the model is only in part relevant to the vascular situation. For instance, dogs fed *n*-3 PUFA-enriched diet displayed early defects in the epithelial regeneration of surgical skin wounds [33]. In addition, dietary supplementation of fish oil *n*-3 PUFA and topical application of the *n*-3 PUFA linolenic acid delayed skin wound healing in humans [34] and rats [35], respectively.

Finally, besides migration, one should take into account other effects of the *n*-3 PUFA on the endothelial cell, such as the interference with pro-inflammatory and-atherogenic programs [36], when evaluating at a cellular level the basis of the beneficial action that the *n*-3 PUFA exert on the vascular function and that is documented by a large body of *in vivo *and clinical studies.

## Conclusions

In conclusion, we have reported here that *n*-3 PUFA inhibit EC migration possibly due to their ability to affect the cytoskeleton. In addition, the observed differences in efficacy between EPA and DHA highlight the importance of the dietary EPA/DHA ratios in clinical practice.

## Methods

### Cell lines, antibodies and reagents

The endothelial cell line H5V, which was derived from the murine heart [37], was cultured in D-MEM (Gibco-BRL) supplemented with 10% fetal bovine serum (Sigma). Mouse anti-vinculin mAb hVIN-1, mouse anti-alpha-tubulin mAb B-5-1-2 and FITC-phalloidin were from Sigma. TRITC-labeled anti-IgG (H+L) anti-mouse antibody was from Jackson Immuno Research Laboratories Inc. The mixture of *n*-3 PUFA ethyl esters (EE) was obtained from SPA (Milan), while the esterified fatty acids EPA and DHA were purchased from ChromaDex and Sigma, respectively. Human fibronectin and the ERK-1/2 inhibitor 2-(2-Amino-3-methoxy-phenyl)-4H-1-benzopyran-4-one (PD098059) were from Sigma. The ERK-1/2 activator N-Arachidonoyl-ethanolamine (anandamide) and the p38/SAPK2 activator 2-(p-Methoxybenzyl)-3,4-pyrrolidinediol-3-acetate (anisomycin) were from Alexis. Antibodies against p44/42 ERK-1/2 and phospho-ERK-1/2 (pThr202/pTyr204) were from Cell Signaling Technology.

### Fatty acid analysis

Membrane isolation and analysis of their relative fatty acid composition were performed as described [38]. Briefly, lipids from approximately 2.5 × 10^5 ^EC were extracted according to the method of Folch [39]. Then, aliquots of the chloroform phases containing lipids were evaporated to dryness. Fatty acid methyl esters in total lipids were prepared and injected into an Autosystem XL gas chromatograph (Perkin Elmer) connected with a flame ionisation detector. Individual fatty acid methyl esters were identified by comparing their retention times with corresponding standards (run in parallel), and their composition was calculated using a Turbochrom system, version 6.1 (Perkin Elmer).

### Wound assay

Cells were seeded (at a density of 1.5 × 10^5^/cm^2^) onto glass cover slips (that had been coated with fibronectin; 7 μg/mL) and grown to confluence. Then, the culture medium was removed, and scratch wounds were produced using a plastic tip for 1,000 μL pipettes. After two washes with D-PBS, cells were incubated with D-MEM medium containing 10% serum, in the presence of either medium alone, or the vehicle (ethanol) or *n*-3 PUFA throughout the migration assay. Finally, cells were fixed with 3.7% paraformaldehyde (for 15 minutes), permeabilized with 0.5% Triton-X-100 (for 3 minutes), blocked with 1% bovine serum albumin (for 60 minutes), stained with FITC-phalloidin and examined by fluorescence microscopy. To quantify migration, the width of the wound was measured using the NIH Image software (version 1.63) [40]. Statistical analysis was performed by Student's T-test, two tailed distribution, two-sample unequal variance.

### Immunofluorescence microscopy

For the analysis of non-contacting cells, cells were seeded onto fibronectin-coated glass cover slips, allowed to adhere for 120 minutes and then incubated with the indicated compound for additional 60 minutes. Cells were then treated as above and stained with primary and TRITC-labeled secondary antibodies, as described [41]. Cover slips were mounted in 488-Mowiol and analyzed with a Zeiss Axiophot microscope (equipped with 100X Plan-Neofluar Ph3 objective lens). A similar protocol was used for analyzing migrating cells at the wound edge.

## List of abbreviations

EC: endothelial cell; EE: ethyl ester; EPA: eicosapentaenoic acid; DHA: docosahexaenoic acid; FA: focal adhesion; MAPK: Mitogen-Activated Protein Kinase; PUFA: Polyunsaturated Fatty Acid

## Competing interests

The authors declare that they have no competing interests.

## Authors' contributions

LT designed the study, carried out the experiments and participated in drafting the manuscript. LM carried out initial immunofluorescence experiments. MTT performed the fatty acid analysis. GB conceived of and coordinated the study and wrote the manuscript. All authors read and approved the final manuscript.
